# Diabetic Ketoacidosis: A Rare Complication of Type 3c Diabetes

**DOI:** 10.7759/cureus.57873

**Published:** 2024-04-08

**Authors:** Jewel Rani Jose, Takshak Shankar, Poonam Arora, Parvathy S, Sreejith Jayachandran

**Affiliations:** 1 Department of Emergency Medicine, All India Institute of Medical Sciences, Rishikesh, Rishikesh, IND

**Keywords:** pancreatic diabetes, type 3c diabetes, pancreatic enzyme replacement therapy, chronic pancreatitis, catecholamine, hyperglycemia, insulin, diabetic ketoacidosis, anion gap

## Abstract

Recently termed pancreoprivic diabetes, type 3c diabetes refers to high blood sugar values secondary to disease of the exocrine pancreas. The disease is most commonly misdiagnosed as type 2 diabetes mellitus (DM) and is overlooked by physicians and the general public. Chronic pancreatitis (CP) accounts for most cases of type 3c diabetes. Diabetic ketoacidosis (DKA) is a rare occurrence in type 3c diabetes as both alpha and beta cell dysfunction occur concurrently. In this case, the major hormones involved in lipolysis and ketone body production would be catecholamines, cortisol, and growth hormone. We report a case of a 37-year-old female with a history of endocrine pancreatic insufficiency secondary to CP who presented with DKA, one of the life-threatening but preventable complications of diabetes. Noncompliance with insulin and concurrent urinary tract infection were the inciting factors. Her condition improved with DKA management according to standard protocol, intravenous antibiotics, and other supportive care. She got discharged after optimization of insulin therapy, with proper advice for home blood sugar monitoring and regular follow-up. A patient with pancreatic pathology may present to the emergency with DKA as the first manifestation, and if not properly evaluated, the diagnosis of type 3c diabetes can be missed.

## Introduction

Hyperglycemia resulting from abnormalities in insulin secretion, insulin action, or both causing metabolic abnormalities is known as diabetes mellitus (DM). Type 3c diabetes, or pancreatogenic diabetes, is a rare form of DM that develops secondary to pancreatic disease or injury. According to the American Diabetes Association (ADA) classification, this includes anatomical and functional loss of glucose-normalizing insulin secretion in the background of exocrine pancreatic dysfunction [1]. The majority of patients who develop type 3c diabetes have a history of chronic pancreatitis (CP), while others have hemochromatosis, cystic fibrosis, pancreatic cancer, fibrocalculous pancreatopathy, pancreatic trauma, pancreatectomy, or pancreatic agenesis [1-4]. In CP, alpha and beta cells of the pancreas are damaged due to cytokine-mediated glandular inflammation and irreversible fibrosis [5]. Diabetic ketoacidosis (DKA) rarely complicates type 3c diabetes, as along with insulin deficiency, these patients also have a concomitant deficiency of glucagon [6].

## Case presentation

A 38-year-old female presented to the emergency department (ED) with a history of multiple episodes of non-bilious, non-bloody vomiting and lower abdominal pain for the past three days. She also reported reduced appetite and malaise for one week. The patient was diagnosed with chronic calcific pancreatitis 11 years ago. Over seven years, she developed exocrine and endocrine pancreatic insufficiency and has been taking insulin and pancreatic enzyme replacement therapy (PERT) since then. She also had a history of multiple common bile duct and pancreatic duct stentings for recurrent abdominal pain. When her symptoms started, she started taking her prescribed medications less frequently.

Upon arrival at the ED, her airway was patent; she had a respiratory rate of 32 breaths per minute, oxygen saturation of 98% while breathing room air, pulse rate of 126 beats per minute, and blood pressure of 112/94 mmHg. She was conscious and oriented. Her breathing pattern was rapid and deep. Signs of dehydration, like dry mucosa and lethargy, were noted. Her systemic examination was normal except for tenderness over the lower abdomen. The initial random blood sugar (RBS) was 549 mg/dL, and urine ketone was largely positive. Blood gas analysis revealed a pH of 7.10, bicarbonate of 4.6 mmol/L, and pCO₂ of 14.1 mmHg. After accounting for albumin, the anion gap was 16.6.

The diagnosis of DKA was made as she had elevated blood sugars, positive urine ketones, and metabolic acidosis. As per standard protocols, treatment with intravenous fluids, potassium supplementation, and insulin infusion was initiated. Urine output and serum electrolytes were monitored during treatment, and proper hydration was ensured. Her abdominal pain and vomiting responded to analgesics and antiemetics. The patient also had features of a urinary tract infection (UTI), and intravenous antibiotics were initiated for the same. After 36 hours, the patient's condition improved, as evidenced by the blood gas showing pH of 7.43, bicarbonate of 20.5 mmol/L, pCO₂ of 25.3 mmHg, and an anion gap of 7.1. Once the patient started tolerating oral feeds, we shifted her to a basal-bolus insulin regime, and blood sugar monitoring was continued. PERT was also re-initiated once the patient started taking oral feeds.

Her HbA1c level was 14.4 mg/dL (4-6.2 mg/dL), and her C-peptide level was 0.13 ng/mL (0.7-1.9 ng/dL). Autoantibodies for islet cells were negative. The complete blood count showed neutrophilic leukocytosis. To look for the precipitating cause of DKA, we did serum lipase levels, cardiac biomarkers, and a complete workup for any possible source of infection. Serum lipase levels and cardiac biomarkers were within normal ranges, and evidence of UTI was present on urine examination. Relevant investigations are tabulated in Table 1. A non-contrast computed tomography of the abdomen was done, and no evidence of complicated UTI or pyelonephritis was found. For our patient, the precipitants were hence noncompliance with medication and infection. The patient was discharged eventually after consultation with the endocrinology department and optimization of insulin therapy. She was given proper advice regarding diet and home blood sugar monitoring. We also emphasized the need for adherence to prescribed treatment and regular follow-up to prevent a recurrence.

**Table 1:** Blood test results. Urine analysis: pH 6.0, leucocytes +, glucose ++++, pus cells 70-80, red blood cells 0-1, and no casts or crystals.

Parameters	Values	Reference range
Hemoglobin	7.8 g/dL	12-15 g/dL
White blood cell count	27.86 × 10³/mcL	4-11 × 10³/mcL
Platelet count	123 ×10³/mcL	150-400 ×10³/mcL
Serum amylase	35 U/L	28-100 U/L
Serum lipase	23 U/L	0-67 U/L
Serum procalcitonin	3.78 ng/mL	0.5-2.0 ng/mL
Serum creatinine	1.10 mg/dL	0.72-1.18 mg/dL
Serum urea	47.0 mg/dL	17-43 mg/dL
Serum sodium	134.0 mEd/dL	136-146 mEq/dL
Serum chloride	102.0 mEq/dL	101-109 mEq/dL
Serum potassium	4.3 mEq/dL	3.5-5.1 mEq/dL
Serum calcium	9.0 mg/dL	8.8-10.6 mg/dL
Total bilirubin	0.33 mg/dL	0.3-1.2 mg/dL
Direct bilirubin	0.07 mg/dL	0-0.2 mg/dL
Aspartate aminotransferase (AST)	19 U/L	0-50 U/L
Alanine aminotransferase (ALT)	14 U/L	0-50 U/L
Alkaline phosphatase (ALP)	266 U/L	30-120 U/L
Serum albumin	2.8 g/dL	3.5-5.2 g/dL
International normalized ratio (INR)	1.10	0.90-1.20

## Discussion

According to Ewald and Bretzel, the major diagnostic criteria for type 3c diabetes include all of the following: pancreatic exocrine deficiency (direct function test or fecal elastase-1 testing), consistent pancreatic abnormalities on imaging (CT, MRI, or endoscopic ultrasound), and the absence of autoimmune markers for type 1 DM. Minor criteria include impaired β-cell function, absence of insulin resistance, low serum concentrations of lipid-soluble vitamins (A, D, E, and K), and impaired incretin secretion [2].

CP develops secondary to inflammation-related glandular fibrosis and atrophy [5]. A well-established correlation exists between decreased β cell mass and insulin secretion in diabetes secondary to CP [7]. Up to 80% of patients may eventually acquire diabetes during their lifetime, and the most important risk factor is disease duration [3]. Data on acute complications such as DKA, hypoglycemia, and target organ damage (retinopathy and nephropathy) associated with type 3c diabetes are limited.

The triad of DKA constitutes hyperglycemia (RBS >250 mg/dL), metabolic acidosis (pH < 7.3 and bicarbonate < 18 mEq/dL), and increased total body ketone concentration (positive serum or urine ketones) [8]. The incidence of DKA ranges from 0 to 56 per 1,000 person-years, with a higher prevalence among women [9]. The common inciting factors for DKA are drug noncompliance, new-onset diabetes, and other acute medical illnesses such as infection, myocardial infarction, pancreatitis, cerebrovascular accidents, and drugs [8-9].

The major anabolic hormone insulin, along with the catabolic hormones glucagon, cortisol, catecholamine, and growth hormone, regulates ketone body production in our body. In the liver, they regulate fatty acid oxidation pathways and the re-esterification process; in the adipose tissue, they regulate fatty acid supply to the liver; and in the periphery, they regulate extrahepatic utilization of ketone bodies [10]. Insulin inhibits lipolysis in adipose tissue and enhances re-esterification. In the liver, it increases fatty acid synthesis and esterification and decreases the transport of fatty acid to the mitochondria [9-10]. Overall, insulin lowers the rate of free fatty acid oxidation and ketogenesis.

Glucagon, in a state of relative insulin deficiency, upregulates enzymes and transporters in the liver, resulting in free fatty acid oxidation and ketogenesis [10]. Cortisol and growth hormones also act in a similar way to glucagon. In 1977, a study was conducted by Barnes et al. in four pancreatectomized subjects and six patients with juvenile diabetes in whom insulin was withheld. The results surprisingly showed that glucagon is not essential for the development of ketoacidosis in diabetes, though it can accelerate the onset of ketonemia and hyperglycemia in the background of insulin deficiency [11].

Catecholamines can stimulate lipolysis and fatty acid production even in the presence of insulin and exert a prominent effect on adipose tissue, with minor effects on the liver and no effect on peripheral utilization [10]. Understanding the role of the insulin-glucagon ratio in type 3c DM is crucial. A distinct finding in type 3c diabetes is that, along with insulin-secreting beta cells, glucagon-secreting alpha cells are also lost. Since the production of both hormones is impaired, the other catabolic hormones mentioned above contribute to the process of ketogenesis. Especially in the event of acute stress, catecholamines might be the dominant hormone involved in lipolysis and ketone body production [6,10]. The exact mechanism of ketogenesis in type 3c diabetes has not been proposed. 

Our patient initially developed exocrine pancreatic insufficiency, which progressed to endocrine pancreatic insufficiency over two years. In CP, the development of exocrine insufficiency typically precedes the development of endocrine insufficiency and type 3c diabetes [5,7]. Treatment of type 3c diabetes is challenging. Metformin is the initial drug advocated, but most patients will eventually need insulin therapy [5]. The role of PERT in this group of patients is inevitable. PERT improves incretin secretion, insulin secretion, and glucose intolerance [4-6]. It also aids in the absorption of fat-soluble vitamins A, D, E, and K [6,12].

## Conclusions

There is a dearth of information in the current literature regarding the pathophysiology of ketone body synthesis in type 3c diabetes. More studies are needed to establish the potential role of catecholamines and further elucidate the hormonal regulation of ketone body production in type 3c diabetes.

In a patient with a history of an insult to the pancreas, recognition of the progression to endocrine insufficiency can be challenging. Loss of regular follow-up with a primary care physician and noncompliance with medication can further worsen the situation. As with any other type of diabetes, patients with type 3c diabetes can present with acute complications like hypoglycemia and DKA. Hence, assessment for DKA and evaluation for precipitating cause should be done in patients presenting with elevated blood sugars, especially in the background of pancreatic pathology. The role of PERT is phenomenal in patients with type 3c diabetes and should be initiated as soon as the diagnosis is made.

## References

[1] (2022). Classification and Diagnosis of Diabetes: Standards of Medical Care in Diabetes-2022. Diabetes Care.

[2] Ewald N, Bretzel RG (2013). Diabetes mellitus secondary to pancreatic diseases (Type 3c)--are we neglecting an important disease?. Eur J Intern Med.

[3] Hart PA, Bellin MD, Andersen DK (2016). Type 3c (pancreatogenic) diabetes mellitus secondary to chronic pancreatitis and pancreatic cancer. Lancet Gastroenterol Hepatol.

[4] Cui Y, Andersen DK (2011). Pancreatogenic diabetes: special considerations for management. Pancreatology.

[5] Andersen DK, Korc M, Petersen GM (2017). Diabetes, pancreatogenic diabetes, and pancreatic cancer. Diabetes.

[6] Melki G, Laham L, Karim G (2019). Chronic pancreatitis leading to pancreatogenic diabetes presenting in diabetic ketoacidosis: a rare entity. Gastroenterology Res.

[7] Schrader H, Menge BA, Schneider S (2009). Reduced pancreatic volume and beta-cell area in patients with chronic pancreatitis. Gastroenterology.

[8] Kitabchi AE, Umpierrez GE, Miles JM, Fisher JN (2009). Hyperglycemic crises in adult patients with diabetes. Diabetes Care.

[9] Lizzo JM, Goyal A, Gupta V (2023). Adult Diabetic Ketoacidosis. http://PMID:32809558..

[10] Alberti KG, Johnston DG, Gill A, Barnes AJ, Orskov H (19781). Hormonal regulation of ketone-body metabolism in man. Biochem Soc Symp.

[11] Barnes AJ, Bloom SR, Goerge K, Alberti GM, Smythe P, Alford FP, Chisholm DJ (1977). Ketoacidosis in pancreatectomized man. N Engl J Med.

[12] Duggan SN, Ewald N, Kelleher L, Griffin O, Gibney J, Conlon KC (2017). The nutritional management of type 3c (pancreatogenic) diabetes in chronic pancreatitis. Eur J Clin Nutr.

